# Twelve-Month Retention in and Impact of Enhance®Fitness on Older Adults in Hawai‘i

**DOI:** 10.1155/2019/9836181

**Published:** 2019-09-02

**Authors:** Michiyo Tomioka, Kathryn L. Braun, Yan Yan Wu, Kay Holt, Paula Keele, Lori Tsuhako, Johnny Yago

**Affiliations:** ^1^Center on the Family, University of Hawai‘i at Mānoa, Honolulu, HI 96822, USA; ^2^Elder Care Services, Kokua Kalihi Valley Comprehensive Family Services, 1846 Gulick Avenue, Honolulu, HI 96819, USA; ^3^Office of Public Health Studies, University of Hawai‘i at Mānoa, Honolulu, HI 96822, USA; ^4^Kaua‘i Agency on Elderly Affairs, Pi‘ikoi Building, 4444 Rice Street, Suite 330, Lihue, Kaua‘i, HI 96766, USA; ^5^Maui County Office on Aging, Aging and Disability Resource Center, Department of Housing and Human Concerns, J. Walter Cameron Center, 95 Mahalani Street, Room 20, Wailuku, HI 96793, USA

## Abstract

**Introduction:**

Enhance®Fitness is a low-cost group exercise program designed specifically for older adults (60+ years) to improve physical performance. The Hawai‘i Healthy Aging Partnership, a statewide health promotion initiative, has continuously offered Enhance®Fitness to Hawai‘i's multicultural population since 2007. This study examined 12-month participation in and impact of Enhance®Fitness on physical performance among older adults in Hawai‘i.

**Method:**

Linear mixed-effects models were applied to analyze the physical performance measures (chair-stands, arm curls, and the up-and-go test) collected at baseline (month 0) and at 4, 8, and 12 months. We also compared the characteristics of participants who participated in the program for 12 months with those who dropped out in order to gain insights on participant retention.

**Results:**

Of 1,202 older adults with baseline data, 427 (35.5%) were continuously enrolled in Enhance®Fitness for 12 months and participated in follow-up data collection. On average, participants attended 63.7% of thrice-weekly classes each month. Participants' physical performance measures improved after 4 months, continued to improve until 8 months, and were maintained thereafter. Besides continuous attendance, performance-measure improvements were associated with younger age, male gender, living with others (vs. alone), and fewer chronic conditions. Compared to those who completed 12 months of the program, the 775 who left the program over the course of the year were more likely to be younger, to be Caucasian (vs. Asian or Pacific Islander), to self-report depression as a chronic condition, and to have lower levels of fitness at baseline. Common reasons for dropping out were illness, relocation, time conflicts, lost interest, and transportation issues.

**Conclusions:**

Long-term participants in Enhance®Fitness initially improved and then maintained physical performance. Future research is needed to identify strategies to maintain enrollment of older adults in the exercise programs over time.

## 1. Introduction

The majority of older adults live with at least one chronic condition that may reduce physical performance or well-being [1, 2]. Poor health and disability are associated with loss of independence and falls. In 2015, the direct medical cost of fall injuries was $50 billion, and it is projected to increase to $67.7 billion by 2020 and to $100 billion by 2030 [2, 3]. Engaging in physical activity is beneficial because it helps not only to prevent falls but also to lower mortality and promote physical and psychological well-being among older adults with and without disabilities [4–10].

Research suggests that participation in physical activity is influenced by a range of personal, social, and environmental factors. Personal factors include age, socioeconomic status, and gender. Social factors include social support, physician influences, and exercise programs such as class size and group cohesion. Environmental factors include availability of free or affordable exercise opportunities and transportation to them [11, 12].

To promote the health of older adults, much attention has been given to implementing health promotion interventions that have proved to be effective in reducing the risk of disability, falls, and chronic conditions [13]. The Hawai‘i Healthy Aging Partnership (HHAP), a statewide health promotion initiative, was formed in 2003 to improve the health of older adults through evidence-based programs [14]. Hawai‘i has a unique population demographic, as two-thirds of the population is of Asian, Native Hawaiian, or other Pacific Islander ancestry. One of the programs implemented has been Enhance®Fitness, a low-cost group exercise program designed specifically for older adults by Sound Generations in Seattle in partnership with the University of Washington [15].

Earlier studies have investigated the impact of Enhance®Fitness on physical performance [15–20]. For example, Belza and colleagues examined 2,889 Enhance®Fitness participants who participated in outcomes testing and found improvements at 4 and 8 months on performance tests [15]. However, no studies outside of Hawai‘i have presented findings on Asian and Native Hawaiian/Pacific Islander (NHPI) elders, and most of the previous studies have examined short-term outcomes [20]. Only one other study was found that examined characteristics of elders who retained by Enhance®Fitness programs and those that drop out. Specifically, in a follow-up survey of elders joining a new Enhance®Fitness site, Gillette and colleagues found that former participants reported lower motivation to exercise and greater barriers than current participants [21]. Thus, this paper fills a gap by presenting findings from a longer-term (12-month) analysis of Enhance®Fitness physical performance data from a sample that includes large proportions of Asians and NHPI elders and adding to the research on program drop out.

## 2. Methods

### 2.1. Intervention

Enhance®Fitness is a low-cost group exercise program offered three times per week for one hour by nationally certified fitness instructors. Each class includes balance, flexibility, cardio, and strengthening exercises. These exercises help participants at any level of fitness by providing adaptations for people with special needs, such as offering seated versions of standing exercises. Each class enrolls up to 25 participants, depending on the space of the site. The classes are offered in a variety of community settings, such as residential and retirement communities, senior housing facilities, adult day care centers, YMCAs, private gyms, churches, and multipurpose centers [15–21].

Among the diverse older adult population of Caucasians, Asians, Native Hawaiian, other Pacific Islanders, and others, some elders adhere strongly to the traditional beliefs and practices of their cultures, while others follow a mix of cultural beliefs and practices. To ensure the attractiveness of and practicality of Enhance®Fitness for Hawai‘i older adults, HHAP worked with the program developers to make minor modifications, such as renaming certain exercises to relate them to daily activity and utilizing culturally appropriate music [22]. The program started with two sites on Kaua‘i County in 2007 (with additional sites added incrementally) and launched in Maui County in 2012.

### 2.2. Study Design

A longitudinal study was conducted. Each participant's physical performance and prevalence of falls were assessed at baseline, which is the first week of the class, and reassessed after 4, 8, and 12 months with the program. Attendance and reasons for drop out were tracked.

### 2.3. Study Sample

Participants aged 50 or older were recruited over time through Area Agencies on Aging and their Aging and Disability Resource Centers (28%), eldercare, faith-based, and healthcare organizations (31%), word-of-mouth (15%), health professionals (2%), and other avenues including community events and mass media (24%). Because participants had to find a way to get to the sites, HHAP partnered with organizations where individuals already congregated or to which they had easy access. For most, family members drop them at the sites, but some utilized public transportation (i.e., bus) or drove themselves. Data were collected from all 19 Enhance®Fitness sites in the state (8 multipurpose centers, 4 senior housing facilities, 5 faith-based organizations, 1 adult daycare, and 1 fitness gym) from 2007 to 2016. At enrollment, participants completed consent, registration, and health history forms and provided physician clearance for participation. No elder was excluded from joining the program, and informed consent was obtained from all participants. This study was approved by Institutional Review Board at the University of Hawai‘i.

### 2.4. Outcome Measures

Physical performance was tested using the Fitness Check created by Sound Generations [23], which adapted standardized measurements by Rikli and Jones [24]. The Fitness Check assesses the participant's lower- and upper-body strength, agility, and balance using three types of tests: chair stand, arm curl, and up-and-go tests. For the chair stand test, the tester records the number of times the participant can move from sitting to standing in 30 seconds. For the arm curl test, the tester records the number of times the participant can perform a bicep curl using a weight (5 pounds for women and 8 pounds for men) in 30 seconds. For the up-and-go test, the observer records the number of seconds it takes for a seated participant to stand, travel 8 feet, round a cone, return to the chair, and be reseated. Participants' attendance was collected each session.

### 2.5. Independent Variables

Independent variables were age group (<65, 65–69, 70–74, 75–79, 80–84, and 85+), gender (female or male), race/ethnicity (Caucasian, Filipino, Japanese, Native Hawaiian and Pacific Islander (NHPI), or other), marital status (married, divorced, widowed, or others), living arrangement (live alone or with others), household income (<15K, 15–25K, 25–50K, 50–75K, or ≥ 75K+), any chronic conditions (e.g., hypertension, arthritis, and depression), and monthly attendance rate. Reasons for drop out were reported by site leaders.

### 2.6. Statistical Analysis

The analysis of baseline data excluded 63 participants because of missing data for age and gender (*n* = 15), chronic conditions (*n* = 11), and/or physical performance measures (*n* = 37). Descriptive statistics were used to summarize the sample characteristics at baseline and to compare the characteristics of those that remained in the program at least 12 months against those that dropped out.

The analysis of 12-month outcomes was limited to elders continuously enrolled for 12 months who participated in the 4-, 8-, and 12-month Fitness Checks. We performed linear mixed-effect model analysis of the trajectories of each physical performance measures (chair stand reps, arm curl reps, and up-and-go seconds) at months 0, 4, 8, and 12 [25]. Random intercepts were used to account for the correlations of repeated measures among each participant and the dependencies of participants nested under the implementation sites. Dispersions of repeated measures were tested but not statistically significant. Independent variables included age group (reference: <65), gender (reference: female), race/ethnicity (reference: Caucasian), marital status (reference: married), living arrangement (reference: alone), household income (reference: <15K), chronic conditions (reference: no conditions), and participation rate. Statistical software R version 3.5.1 was used to analyze the data. The data that support the findings of this study are available from the corresponding author, MT, upon reasonable request.

## 3. Results

Between 2007 and 2016, 1,202 older adult participants enrolled in Enhance®Fitness and completed baseline data collection. Not all enrollees choose to continue long-term with the program, with 546 lost by the 4-month follow-up, another 194 lost by the 8-month follow-up, and another 35 lost by the 12-month follow-up. The remaining 427 (35.5%) participants were continuously enrolled for 12 months and participated in the 4-, 8-, and 12-month Fitness Checks; analysis is limited to this group. Of these, 125 (29.3%) were Caucasian, 46 (10.8%) were Filipino, 198 (46.4%) were Japanese, 32 (7.5%) were NHPI, and 26 (6.1%) were from other ethnic groups (Table 1). The majority was 65 years or older (85.7%) and female (90.2%). More than half of them lived with others (68.1%) and had at least one chronic condition (74.2%). Nearly half of them were married (49.6%) and had an annual household income of less than 25K (40.3%). While in the program, participants attended 63.7% (about 8) of the 12–13 classes offered each month. This equates to about 100 minutes of moderate exercise per week.

The 775 individuals who discontinued their participation dropped out over time. Based on reports by site leaders, the most common reasons for stopping, in order of frequency, were illness, time conflicts, relocation, lost interest, and transportation issues. An examination of baseline data suggests that those that dropped out differed from those that stayed in several variables. A significantly larger proportion of younger participants dropped out than older participants. Caucasians were more likely to drop out, and Japanese were more likely to stay in the program, while the proportions of the other ethnic groups remained constant. The groups did not differ in number of chronic conditions; however, a significantly greater proportion of drop outs reported depression (11.5% vs. 6.6%). The groups also differed in baseline fitness measures, with the drop outs having lower levels of fitness. Those that dropped out over the course of the year also attended a significantly lower percentage of available classes than those that continued. Although gender was not a significant predictor of drop out, we found that males had better performance measures than females at baseline (not shown in table). There were no differences between the groups based on being married, living alone, or household income.

Table 2 shows the multivariate results of physical performance tests. The mean number of chair stands increased by 2.32 repetitions (95% confidence interval (CI): 1.99 to 2.65; *p* < 0.0001) at 4 months and by an additional 1.2 repetitions (95% CI: 0.84 to 1.56; *p* < 0.0001) at 8 months, and changes were maintained at month 12. Among the control variables, age and gender were associated with average number of repetitions, with males showing more improvement than females and individuals <65 years showing more improvement than individuals aged 70 and older. Other associated variables included marital status (married participants improved more than nonmarried participants), living arrangement (those living with others improved more than those living alone), and presence of chronic conditions (those with no chronic conditions made greater improvement).

In multivariate analysis, the mean number of arm curls increased by 2.20 repetitions at 4 months (95% CI: 1.85 to 2.54; *p* < 0.0001), an additional 0.95 repetitions at 8 months (95% CI: 0.57 to 1.32; *p* < 0.0001), and an additional 0.52 repetitions at 12 months (95% CI: 0.16 to 0.89; *p* < 0.0051). Other variables associated with arm curls in the analysis were age and gender, with males and participants <65 years showing greater improvement.

The number of seconds to complete the up-and-go test decreased by 0.85 seconds at 4 months (95% CI: 0.67 to 1.03; *p* < 0.0001) and another 0.24 seconds at 8 months (95% CI: 0.05 to 0.44; *p* < 0.0001). Control variables associated with improvement included age (with less improvement in individuals age 75 and older), marital status (with greater improvement for those married), living arrangements (with less improvement in those living alone), and presence of chronic conditions (Table 2).

**Figure 1:**
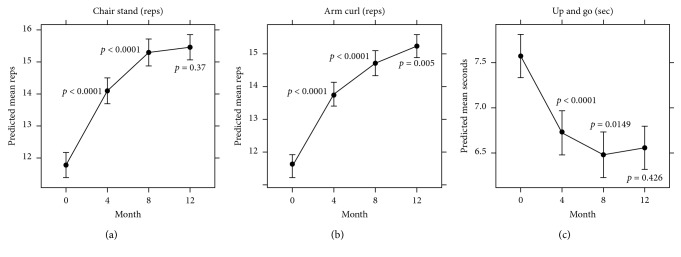
Trajectories of mean performance measures and 95% confidence intervals (CIs) at baseline, 4, 8, and 12 months. Statistical significance for the test of changes in physical performance measures at each follow-up month (compared with the previous visit) is indicated by *p* values.

As illustrated in Figure 1, participation in Enhance®Fitness was associated with improvement at 4 months and 8 months in all three physical performance measures, with maintenance of these gains at 12 months.

## 4. Discussion

In this longitudinal study of Enhance®Fitness in Hawai‘i, participants' physical performance measures improved between baseline and the 4-month and 8-months measures, and participants' higher physical performance levels were maintained at 12 months.

Improvements in physical performance were also found in multiple previous Enhance®Fitness studies, as shown in the scoping review of qualitative and quantitative studies conducted by Petrescu-Prahova and colleagues [20]. Other studies show its effectiveness with minority populations [15, 17, 18] and at a variety of Enhance®Fitness sites [16]. More specifically, most studies of Enhance®Fitness have observed positive physical performance findings after 4 to 8 months with the program [15, 16, 18, 20, 22]. This study adds to the literature by examining the longer-term impact of Enhance®Fitness in an Asian and NHPI population, controlling for baseline demographic and health variables.

As seen in previous studies, physical performance was significantly associated with age, chronic conditions, living arrangements, and social support, as well as participation in a physical activity program. For example, findings from a longitudinal cohort study in Taiwan found less functional improvement among older adults not living with spouses and with low social support [26]. A prospective cohort study of community-dwelling older Canadians found that decreased fitness and increased frailty were associated with greater age, poorer health, more comorbid conditions, and greater social isolation [27]. We also found that males had better performance measures than females at baseline and that gender was a significant covariate in the linear mixed-effects model of improvement for the chair-stand and arm-curl measures.

The findings also provide insights into long-term participation in exercise. We found that younger adults were more likely to drop out than older adults, perhaps suggesting that younger adults had more options for socialization and exercise and did not feel a need to continue. In this Hawai‘i-based sample, Japanese were more likely to maintain participation in the program than Caucasian. This is not a surprising finding in Hawai‘i, where Japanese have better health than Caucasians. For example, smaller proportions of Japanese elders are overweight or obese (44%) compared to Caucasian elders (57%), and life expectancy for Japanese is four years longer than for Caucasians in the state [28, 29]. Older Caucasians also may be temporary (winter) visitors to Hawai‘i from other states who join Enhance®Fitness on a short-term basis or may be more likely that other groups to relocate to the other states in old age to be closer to family and/or to escape the high cost of living.

A 2014 review of literature on older adult adherence to exercise programs found better adherence in older adults with higher socioeconomic status, fewer chronic conditions, better physical abilities, and fewer depressive symptoms [30]. In our study, baseline physical fitness was associated with adherence, with sustainers having higher fitness levels at baseline. Also, a greater proportion of dropouts reported depression. This is unfortunate finding, as group exercise programs could have antidepressive qualities. Research suggests that physical activity releases endorphins, which can increase feelings of well-being and enhance self-efficacy and self-esteem [31]. Group exercise has the additional potential benefit of increasing opportunities for socialization and friendship, thus a potential mitigation of social isolation.

Despite successful outcomes associated with long-term participation in Enhance®Fitness, further research is needed on why older adults drop out and strategies to keep them engaged. Burton and colleagues surveyed older adults on reasons for dropping out of a resistance training program. Half of respondents reported dropping out within 4 months of enrollment, and the most common reasons were injury, illness, holidays, and the feeling that the program was not suitable for them [32]. Several researchers have published on reasons and motivators of adults to continue in exercise trials. For example, Buchholz and colleagues found that women with more hardships and perceived poor health took more reminder phone calls to encourage their continued participation [33]. In a study of motivators to join and stay with an exercise trial, Viljoen and Christie found that participant motivators changed over time. Initially, participants were attracted to the exercise trial, because they wanted to join a structured exercise program. However, reasons for staying with the trial included a commitment to the researcher and the social aspects of the group exercise program [34]. These offer promising clues for future research.

There are several limitations of this study. First, participant data were collected by instructors and program staff. Although they were trained to correctly administer the Fitness Check, deviations over time and across the 19 sites may have occurred. Second, we did not have a control group for this study. All the participants in this study received medical clearance and chose to participate and participated as long as they desired.

Despite limitations, this study contributes to demonstrating the positive long-term impact of Enhance®Fitness on physical performance as well as risk factors associated with dropping out of an exercise program. Although no amount of physical activity can stop the biological aging process, regular participation in exercise increases active life expectancy by slowing physiological decline and development and progression of chronic disease and disabling conditions [4]. Health professionals and gerontologists who consider implementing evidence-based program should plan ways to sustain programs over the long term, educate elders and policy makers on the importance of exercise continuity, and develop strategies to keep participants, especially those with depression, in the program. Exercise programs, especially evidence-based programs like Enhance®Fitness, that can attract a variety of older adults, are a worthy investment.

## Figures and Tables

**Table 1:** Characteristics of the full sample (*N* = 1,202), those completing 12 months (*N* = 427), and those dropping out (*N* = 775).

	Baseline (*N* = 1,202), *N* (%)	12 months (*N* = 427), *N* (%)	Dropped-out (*N* = 775), *N* (%)	Significance probability
Baseline age				
<65	217 (18.1)	61 (14.3)	156 (20.1)	*p* < 0.001
65–69	269 (22.4)	91 (21.3)	178 (23.0)	
70–74	225 (18.7)	69 (16.2)	156 (20.1)	
75–79	193 (16.1)	83 (19.4)	110 (14.2)	
80–84	155 (12.9)	74 (17.3)	81 (10.5)	
85+	143 (11.9)	49 (11.5)	94 (12.1)	
Female gender	1053 (87.6)	385 (90.2)	668 (86.2)	ns
Race/ethnicity				
Caucasian	474 (39.4)	125 (29.3)	349 (45.0)	*p* < 0.001
Filipino	153 (12.7)	46 (10.8)	107 (13.8)	
Japanese	369 (30.7)	198 (46.4)	171 (22.1)	
NHPI	101 (8.4)	32 (7.5)	69 (8.9)	
Others	105 (8.7)	26 (6.1)	79 (10.2)	
Married	574 (47.8)	212 (49.6)	362 (46.7)	ns
Living alone	401 (33.4)	136 (31.9)	265 (34.2)	ns
Household income				
<15K	247 (20.5)	73 (17.1)	174 (22.5)	ns
15–25K	267 (22.2)	99 (23.2)	168 (21.7)	
25–50K	291 (24.2)	107 (25.1)	184 (23.7)	
50–75K	154 (12.8)	55 (12.9)	99 (12.8)	
75K+	97 (8.1)	44 (10.3)	53 (6.8)	
Unknown	146 (12.1)	49 (11.5)	97 (12.5)	
Chronic condition(s)	888 (73.9)	317 (74.2)	571 (73.7)	ns
Depression	117 (9.7)	28 (6.6)	89 (11.5)	*p*=0.008
Performance measure (baseline)	Mean (SD)	Mean (SD)	Mean (SD)	
Chair stands	11.3 (3.9)	11.6 (4.0)	11.1 (3.9)	*p*=0.05
Arm curls	12.0 (4.1)	11.7 (4.0)	12.2 (4.3)	*p*=0.01
Up-and-go	7.8 (4.2)	7.7 (3.6)	8.0 (4.7)	*p*=0.05
Participation (%)	55.8 (18.5)	63.7 (14.6)	51.4 (19.0)	*p* < 0.001

**Table 2:** Mean differences (Est) in physical performance tests (chair stand, arm curl, and up-and-go) and 95% confidence intervals (CIs) estimated from multivariate linear mixed-effects models.

	Chair stand (repetitions)			Arm curl (repetitions)			Up and go (seconds)		
	Est	95% CI	*p* value	Est	95% CI	*p* value	Est	95% CI	*p* value
Intercept	16.05	(14.27, 17.84)	<0.0001	14.34	(12.83, 15.85)	<0.0001	3.63	(2.50, 4.75)	<0.0001
Month (ref: month 0)									
Change 0–4	2.32	(1.99, 2.65)	<0.0001	2.20	(1.85, 2.54)	<0.0001	−0.85	(−1.03, −0.67)	<0.0001
Change 4–8	1.20	(0.84, 1.56)	<0.0001	0.95	(0.57, 1.32)	<0.0001	−0.24	(−0.44, −0.05)	0.0149
Change 8–12	0.16	(−0.19, 0.52)	0.3662	0.52	(0.16, 0.89)	0.0051	0.08	(−0.11, 0.27)	0.4260
Age group (ref: <65)									
65–69	−0.98	(−2.18, 0.22)	0.1100	−0.38	(−1.39, 0.63)	0.4558	0.31	(−0.45, 1.06)	0.4287
70–74	−2.14	(−3.44, −0.83)	0.0013	−1.71	(−2.81, −0.60)	0.0024	0.98	(0.16, 1.80)	0.0196
75–79	−3.69	(−4.96, −2.42)	<0.0001	−2.15	(−3.22, −1.08)	<0.0001	1.64	(0.84, 2.44)	<0.0001
80–84	−4.55	(−5.90, −3.19)	<0.0001	−3.80	(−4.94, −2.67)	<0.0001	3.04	(2.19, 3.89)	<0.0001
85+	−6.30	(−7.81, −4.79)	<0.0001	−4.34	(−5.61, −3.07)	<0.0001	4.08	(3.13, 5.03)	<0.0001
Gender (ref: female)									
Male	1.71	(0.48, 2.94)	0.0063	1.57	(0.53, 2.60)	0.0030	−0.31	(−1.08, 0.46)	0.4350
Race/ethnicity (ref: white)									
Filipino	0.77	(−0.56, 2.09)	0.2571	−0.67	(−1.79, 0.45)	0.2405	0.51	(−0.33, 1.35)	0.2313
Japanese	1.93	(1.04, 2.83)	<0.0001	−0.43	(−1.18, 0.33)	0.2692	−0.28	(−0.84, 0.29)	0.3385
NHPI	0.37	(−1.07, 1.81)	0.6141	0.27	(−0.94, 1.48)	0.6598	0.49	(−0.41, 1.40)	0.2849
Others	−0.20	(−1.79, 1.38)	0.8000	−0.30	(−1.64, 1.04)	0.6637	1.04	(0.05, 2.04)	0.0397
Marital status (ref: married)									
Divorced	−1.76	(−3.24, −0.29)	0.0188	−0.59	(−1.83, 0.65)	0.3500	1.24	(0.31, 2.17)	0.0086
Widowed	−1.15	(−2.19, −0.10)	0.0322	−1.15	(−2.04, −0.27)	0.0106	1.86	(1.20, 2.52)	<0.0001
Others	−2.89	(−4.19, −1.59)	<0.0001	−1.06	(−2.15, 0.04)	0.0587	1.21	(0.39, 2.02)	0.0038
Living arrangement (ref: live alone)									
With others	−1.57	(−2.56, −0.59)	0.0018	−0.79	(−1.63, 0.04)	0.0618	1.57	(0.95, 2.19)	<0.0001
Household income (ref: <15K)									
15–25K	0.46	(−0.68, 1.59)	0.4302	0.69	(−0.27, 1.64)	0.1576	−0.04	(−0.76, 0.68)	0.9106
25–50K	−0.67	(−1.80, 0.47)	0.2486	0.45	(−0.50, 1.40)	0.3543	0.31	(−0.41, 1.02)	0.4009
50–75K	0.59	(−0.75, 1.92)	0.3908	0.91	(−0.21, 2.04)	0.1102	−0.29	(−1.13, 0.55)	0.4947
75K+	−0.55	(−2.03, 0.94)	0.4729	0.55	(−0.70, 1.80)	0.3885	−0.51	(−1.45, 0.43)	0.2867
Unknown	−0.72	(−2.10, 0.66)	0.3038	−0.20	(−1.35, 0.96)	0.7391	0.54	(−0.33, 1.40)	0.2255
Chronic disease (ref: no)									
Yes	−0.88	(−1.70, −0.06)	0.0354	−0.04	(−0.73, 0.65)	0.9085	0.76	(0.24, 1.27)	0.0040
Percent of participation									
1% increase	−0.01	(−0.03, 0.02)	0.6377	0.00	(−0.02, 0.02)	0.7988	−0.01	(−0.03, 0.00)	0.1770

## Data Availability

The data used to support the findings of this study are available from the corresponding author upon request.
